# Photoformation of Environmentally Persistent Free Radicals During Phototransformation of Poly-Cyclic Aromatic Hydrocarbons (PAHs) on Particles in an Aqueous Solution: The Hydrogenation of PAHs and Effect of Co-Existing Water Matrix Factors

**DOI:** 10.3390/toxics12110796

**Published:** 2024-10-31

**Authors:** Xintong Li, Baocheng Qu, Jingyao Wang, Hongxia Zhao

**Affiliations:** 1 Key Laboratory of Industrial Ecology and Environmental Engineering (Ministry of Education), School of Environmental Science and Technology, Dalian University of Technology, Linggong Road 2, Dalian 116024, China; llxt@mail.dlut.edu.cn (X.L.); wangjingyao1012@mail.dlut.edu.cn (J.W.); 2 College of Marine Technology and Environment, Dalian Ocean University, Dalian 116023, China; qubaocheng@dlou.edu.cn; 3 Key Laboratory of Environment Controlled Aquaculture, Ministry of Education, Dalian 116023, China

**Keywords:** environmentally persistent free radicals, photodegradation, hydrogenation of polycyclic aromatic hydrocarbons, EPR

## Abstract

Environmentally persistent free radicals (EPFRs) generated on particles under irradiation in water have attracted particular attention, and their formation mechanisms are not well understood. This study investigated the photoformation of EPFRs on both actual samples collected from an oil production plant in Panjin, Liaoning, China, and simulated Fe(III)-montmorillonite samples in water. The EPFRs detected on actual samples were not easily generated compared with those in the soil or in the air, based on the concentrations of identified PAHs. EPR signals in the range of 10^17^ to 10^18^ spin/g were detected on the simulated Fe(III)-montmorillonite samples. Their g factors were smaller than 2.0030, which indicated the generation of carbon-centered EPFRs. The primary byproducts were identified by chromatography–mass spectrometry (GC-MS), and a possible EPFR formation pathway during PAH degradation was proposed. Hydrogenation of PAHs during the photoformation of EPFRs was observed and might be due to the catalysis of the simulated particles and the interaction of the intermediates. Meanwhile, the effects of the typical anions (NO_2_^−^ and Cl^−^) and the surfactant (TWEEN^®^ 80 and sodium dodecyl sulfate) were investigated and indicated that the phototransformation process and adsorption process would affect the formation of EPFRs. Overall, our study provided useful information to understand the photoformation of EPFRs in aqueous environments.

## 1. Introduction

Environmentally persistent free radicals (EPFRs) are defined as organic free radicals with long lifetimes stabilized on or inside particles [1,2]. In recent years, studies have found the presence of EPFRs in soil particles [3,4,5], fly ash [6,7], atmospheric particulates [8,9,10,11], biochar [12,13,14], and microplastic particles [15,16,17]. The adsorption and stabilization of these particles during the formation of EPFRs could effectively protect EPFRs from rapid oxidation and prolong their lifetimes to tens of hours or days [18]. Like the traditional free radicals, the unpaired electron in EPFRs could induce the generation of reactive oxygen species, which have high reactivity and would cause biological damage through oxidative stress in cells. Considering the wide distribution in the environment, the formation mechanism of EPFRs on these particles has attracted increasing research interests and the original transformation pathway of the organic precursors might change. The phototransformation of catechol to the dimer-type products during EPFR generation under UV irradiation on transition metal oxide particles has been reported [19]. The decomposition of surface-bound phenoxyl radicals in a gas phase through CO elimination could generate cyclopentadienyl radicals [20]; these transformation pathway changes often occur in the gas phase reactions of organic precursors or nanoscale particulate systems simulated in a laboratory. The literature lacks detailed investigation of the phototransformation pathway change during EPFR formation in a real environment. Considering that the phototransformation of organic contaminants into harmful and toxic byproducts in water could be further affected during EPFR formation [21,22], it is necessary and significant to study the relationship between the transformation of these organic contaminants and EPFR formation, which could provide new insights into the elimination of the environmental concerns regarding organic pollutants in water.

Polycyclic aromatic hydrocarbons (PAHs) are a kind of ideal organic precursor for EPFR formation with delocalized π-electrons in an aromatic ring [23]. They are known to readily absorb visible light and UVA [24]. UVA absorption leads to the formation of reactive intermediate species, releasing large amounts of energy, and could improve the reactivity of PAHs [25]. The subsequent transformation of these intermediates is considered to be key to the conversion into EPFRs [26,27]. It has been reported that the hydroxylation and oxidation of anthracene, pyrene, benzo[a]pyrene, and benzo[a]anthracene resulted in the formation of EPFRs on particles [23,28,29,30]; transforming PAHs into less toxic compounds involving their reductive hydrogenation would be an effective technique to detoxify them and has been studied extensively [31,32]. The systematic investigation at the density functional theory level of the PAHs showed trends for bonding hydrogen [33]. Particles with transition metal ions could act as effective catalysts for reductive hydrogenation reactions and have been demonstrated to function well in aqueous solutions such as those of groundwater environments [34,35]. The hydrogenation of PAHs might also occur during EPFR formation in water. As a new transformation pathway during EPFR formation, increasing the understanding of their formation mechanism would encourage more researchers to focus on preventing and controlling PAHs.

The factors affecting EPFR formation have attracted wide attention. As previously reported, the formation of EPFRs could be affected by particle properties (size, metal redox capacity, etc.) and reaction conditions (temperature, light intensity, etc.) and could be ascribed to electron transfer capacity, particle adsorption capacity, and the organic precursor transformation rate [8,36,37,38]. In water, a large number of co-existing water matrixes might also affect the EPFR formation [22,39]. Typical anions, such as chloride (Cl^−^) and nitrite (NO_2_^−^), are common components in natural water and are easy to transfer to excited states under irradiation, which further affect the indirect photolysis of organic precursors [40,41,42]. As for the surfactants from emissions, their interaction with the surface of particles would affect the interfacial tension and the adsorption capacity of the particles [43,44], which in turn might have an impact on EPFR formation. At present, only a few research studies on the effect factors of EPFR formation in water have been reported. It is vital to understand their influencing mechanisms as this will be helpful in controlling and preventing the sources of EPFRs in water.

In the present study, to understand the characteristics and mechanisms of the photoformation of EPFRs in an aqueous solution, we adopted both field investigation and lab simulation to study EPFR occurrence. The dynamics, types, and decay of photochemically generated EPFRs on actual particles from PAH-contaminated sites were systematically studied using an electron paramagnetic resonance (EPR) spectrometer. The characteristics of EPFRs generated by the representative PAHs on simulated Fe(III)-montmorillonite samples were also compared, and the possible formation mechanism was discussed. Finally, this study investigated the effects of potential co-existing water matrix factors on EPFR formation. The important conclusion of this study was to demonstrate that the hydrogenation of PAHs was an important route for the photogeneration of EPFRs in water and that it was most likely due to the interaction of particles with the intermediates and the interference of the water environment. This study may provide a new perspective on the source of EPFRs in an aqueous environment and the risk assessment of PAHs.

## 2. Methods

### 2.1. Chemicals and Reagents

Naphthalene (Nap ≥ 99.7%), fluorene (FLU ≥ 99%), fluoranthene (FLA ≥ 98%), pyrene (PYR ≥ 99%), benzo[a]anthracene (BaA ≥ 98%), Benzo[b]fluoranthene (BbF ≥ 95%), Benzo[g–i]perylene (BghiP ≥ 97%), anhydrous ferric chloride (FeCl_3_ ≥ 99.9%), sodium nitrite (NaNO_2_ ≥ 99.99%), sodium chloride (NaCl ≥ 99.5%), TWEEN^®^ 80, sodium dodecyl sulfate (SDS, CH_3_(CH_2_)_11_OSO_3_Na ≥ 98%), and 2,2-Diphenyl-1-picrylhydrazyl (DPPH) standard were all purchased from Aladdin Co. Ltd. (Shanghai, China). Other target standards and surrogate standards are presented in Text S1. Montmorillonite was purchased from Aladdin Co. Ltd. (Shanghai, China), and the ultrapure water (18 MΩ) used in all the experiments was prepared by an OKP Ultrapure Water System (Shanghai Lakecore Instrument Co.; Shanghai, China). Dichloromethane, acetone, and n-hexane were of HPLC grade and were provided by Sigma-Aldrich (St. Louis, MO, USA).

### 2.2. Sample Collection and Preparation

All the actual particle samples were collected from an oil production plant (121°43’ E; 31°02’ N) in Panjin, Liaoning, China, on 11 July 2020. The sampling sites were located at a distance of 500 m in four directions (east, west, south, and north) from the plant. Five surface samples (0–5 cm in depth and 200 g each) were collected from each site and mixed together. The samples were ground and sieved by a 200-mesh sifter to eliminate any coarse-sized minerals and plant roots. The simulated samples were prepared using Fe(III)-montmorillonite particles, considering that montmorillonite is common in the environment as a representative clay mineral with a good adsorption ability, and Fe(III) ions could play an important role in electron transfer [30,38,45]. The preparation method is given in detail in our previous paper [46] and Text S2. All the samples were stored at −4 °C until used for further experiments.

### 2.3. Irradiation Experiments

Fifty milligrams of both the actual samples and the simulated Fe(III)-montmorillonite samples were mixed with 20 mL of solution and irradiated for 4 h. The difference was that water was directly added into the photolysis tubes of the actual samples, while solutions with 7 kinds of PAHs were separately added to the simulated samples. The concentration of the 7 kinds of PAHs was uniformly set to 50 mM (adding acetone as a cosolvent). Irradiation experiments were performed in an XPA-1 photochemical reactor (Xujiang, Nanjing, China) equipped with 290 nm cut-off filters and a circulation of cold water. A 1000 W Xe lamp was chosen as the light source, whose light intensity was about 12.89 mW/cm^2^ (by optical sensor, RAMSES, TriOS, Rastede, Germany). Dark controls and parallel controls were both considered under the same irradiation conditions. After photoreaction, the samples were immediately taken out for further analysis under centrifugation at a 3500 g speed and desiccation. In addition, the reaction solution was supplemented with certain concentrations of NaCl, NaNO_2_ (0.02 mol/L and 0.2 mol/L) and TWEEN^®^ 80 (1% and 5% in volume), and sodium dodecyl sulfate (0.25 g/L and 2.5 g/L) to investigate their effects on EPFR formation.

### 2.4. Electron Paramagnetic Resonance Spectroscopic Characterization and Gas Chromatograph/Mass Spectrometer Analysis

Samples weighing 20 mg were placed into high-purity quartz EPR tubes (4 mm, Norell, Landisville, NJ, USA) and analyzed at room temperature using a Bruker A200 electron paramagnetic resonance spectrometer (Karlsruhe, Germany). The EPR signals were calculated as spins based on the standard curve and normalized by sample weight with the help of DPPH. The instrument parameters, operation, and quantification of the EPFRs are presented in Text S3.

The information about the actual sample and the simulated Fe(III)-montmorillonite sample extractions is presented in Text S4. The concentrations of the PAHs were determined using a GC-MS system equipped with a single-quadrupole mass analyzer (Shimadzu, GCMS-QP2020, Kyoto, Japan), operated in the selected ion monitoring (SIM) mode. The chromatographic column was an SH-RXI-5sil MS column (0.25 mm i.d. ×30 m, 0.25 μm film thickness) and was utilized to separate the PAHs using high-purity helium as the carrier gas at a flow rate of 1 mL/min. The thermal gradient was set as follows: 80 °C for 3 min, increased to 290 °C at 5 °C/min, and held for 5 min. The injector and ion source temperatures were separately set at 290 °C and 230 °C. The photoconversion byproducts were identified using a Q Exactive GC Orbitrap GC-MS/MS (Thermo Fisher Scientific, 2021AAEE, Waltham, MA, USA). More details are shown in Text S5.

## 3. Results and Discussion

### 3.1. Photoformation of EPFRs on Actual Particles in an Aqueous Solution

**Figure 1:**
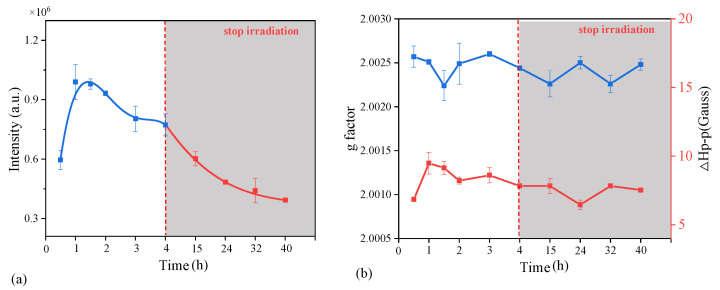
Photoformation and decay of EPFRs on actual particles in water: (**a**) EPR signal intensity (a.u.) trend of actual samples during the 4 h irradiation and 40 h of laying aside (the linked lines aim to help show the trend); (**b**) trends of g value (the blue line) and line width (ΔH_p-p_) of EPR signals (the red line).

We directly irradiated the samples collected from the oil production plant in pure water with no additions to study the photoformation of EPFRs on actual particles. The significant EPR signals were observed (Figure S1) after irradiation, and it could be speculated that irradiation was one of the indispensable conditions of EPFR formation in water. As shown in Figure 1a, the intensity trends of the EPFRs were shown during the irradiation for 4 h; then, they were laid aside for 40 h in the dark. The EPFRs were quickly generated during irradiation for 1 h and then gradually decreased over the next 3 h. After the irradiation stopped, a continuous reduction in EPR signal intensity was observed over the next few dozens of hours. These trends were similar to the results of our previous study using actual particles in anthracene solution, and the explanation was also given [46]. Additionally, a stable trend of the g value and line width (ΔH_p-p_) of the EPR signals was shown at the average of 2.0025 and 7.97 Gauss, respectively, during the irradiation and dark time (Figure 1b). These results indicated that the type of EPFRs was basically unchanged and corresponded to the characteristics of carbon-centered radicals whose g value is usually less than 2.003 [47]. The half-life time of the EPFRs was also calculated by the dynamic changes in the intensity and was more than 38.01 h. The half-life time of tens of hours was not as long as those of the EPFRs reported on soil particles or atmospheric particulate matter, with hundreds of hours or tens of days; this could be explained from two perspectives: (i) The EPFRs types were different and a semiquinone-type radical was less likely to decompose than carbon-centered radicals, resulting in a slow decay of lifetime [48,49]; (ii) water might not be conducive to the stability of EPFRs and may accelerate the decay of EPFRs on the particles [1].

As has previously been discussed, the PAH molecules were involved in the formation of EPFRs due to their highly delocalized π-electrons and the possibility of electron transfer. Based on various reports on PAHs as organic precursors of EPFRs, we measured the PAHs of the collected samples in this region. The concentrations of 13 identified PAHs are listed in Table S1 (FLA, PHE, and CHR were not detected); their pollution levels were at μg/g, and the sum concentrations of these samples were in the range of 1520.37–2539.23 μg/g (dw). Therein, the high molecular weight PAHs (4–6 rings) dominated and low molecular weight PAHs (2–3 rings) took a smaller proportion. Considering that their sum concentrations were much higher than the mean value of the 16 total PAH concentrations from the soil in China, and ranged from 0.0037 to 23.300 μg/g (dw) [50], other control groups, using lab-prepared aging particles with a relatively low concentration of PAHs, were irradiated and detected. Although it has been confirmed that soil particles and atmospheric particulate matter with similar concentrations of PAHs could generate EPFRs (Table S2), no EPFRs were detected in these control groups with a low concentration of PAHs (Figure S2), indicating that EPFRs are not easily generated in an aqueous solution compared with those in soil or in air; consequently, more organic precursors were needed. Our results suggest that the high level of contaminated actual particles could still be a new source of EPFRs photogenerated in water.

### 3.2. Photoformation of EPFRs During Different PAHs Degradation

**Figure 2:**
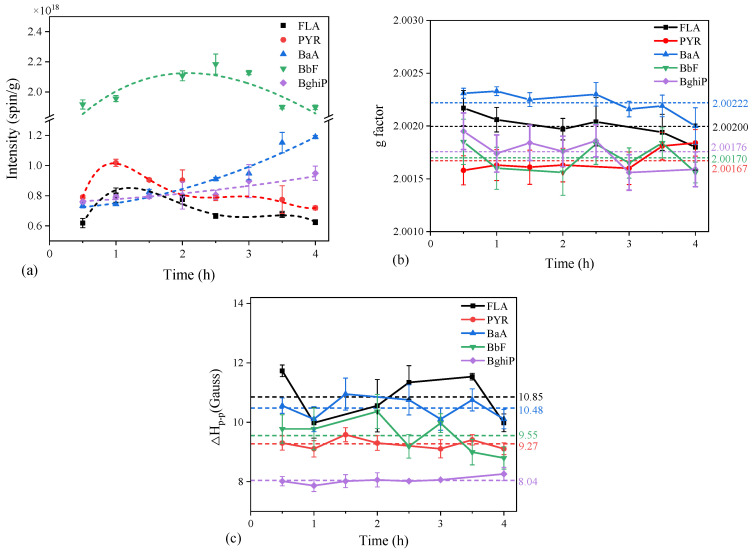
Photoformation of EPFRs on Fe(III)-montmorillonite particles in water: (**a**) EPR signal intensity (spin/g) trend during the 4 h irradiation (the linked lines aim to help show the trend); (**b**) trends of g factor of the simulated samples; (**c**) trends of line width (ΔH_p-p_) of EPR signals.

It was proposed that the PAH degradation may be involved in the photoformation process of EPFRs [36,51]. The simulated Fe(III)-montmorillonite particles were used as a clean surrogate of actual particles, and seven model PAHs (Nap, Flu, FLA, PYR, BaA, BbF, and BghiP) were selected based on the concentration and number of rings in the actual samples to test this hypothesis and exclude the influence of the complexity of the actual samples, such as the dissolved matter and various kinds of transition metal ions [52,53,54,55]. As shown in Figure 2, the FLA-EPFRs (three rings), PYR-EPFRs (four rings), and BbF-EPFRs (four rings) showed a tendency to increase and then decrease during the irradiation, which was similar to those on the actual samples. As for the BaA-EPFRs (four rings) and BghiP-EPFRs (six rings), their signal intensity continued to increase during the irradiation. On the other hand, the characteristics (g value and ΔH_p-p_) were also investigated and reflected relatively stable trends, and the g values were all less than 2.0030, which further verified the formation of carbon-centered EPFRs on the actual samples, as mentioned. The signals of the FLA-EPFRs (three rings) and BaA-EPFRs (four rings) showed a larger g value and ΔH_p-p_, while Nap-EPFRs and Flu-EPFRs were not detected (Figure S2). In conclusion, the EPFRs generated by these PAHs showed a certain difference in the generation trends and characteristics (g value or ΔH_p-p_). We speculated that the phototransformation pathways and the structures of the intermediates of these PAHs were closely related to the photoformation of the EPFRs when ignoring the influence of particle properties and their electron transfer ability.

**Figure 3:**
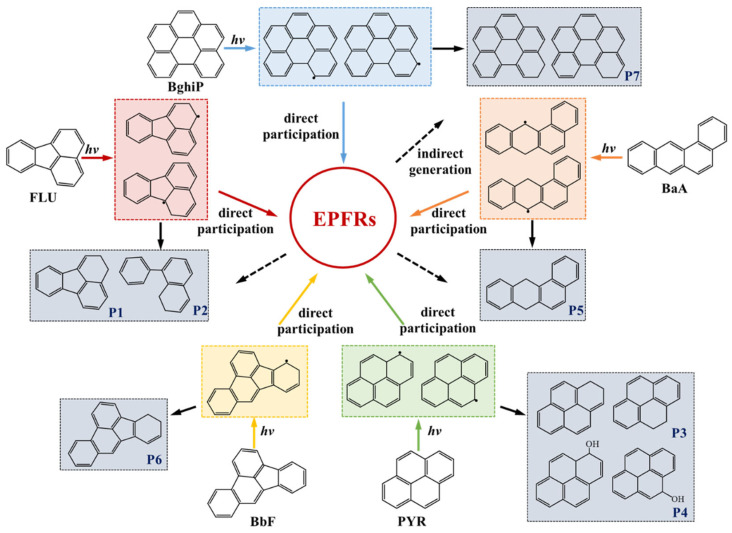
Possible reaction scheme for EPFR formation during the photodegradation of PAHs under irradiation in water.

To further clarify the possible reaction scheme in the PAH-Fe(III)-montmorillonite system, various degradation byproducts were detected. Four familiar methods based on the electronic structure of PAHs, such as the highest occupied molecular orbital (HOMO) compositions, population of *π* electrons (pp-*π*), Fukui function (FF), and dual descriptor (DD) were used to predict the reactivity of PAHs under irradiation and to guide the determination of byproducts [56]. The structures and relevant numberings and the substitution sites of the reactants are listed in Table S3. The most reactive sites were judged based on comparisons of these methods, and the intermediates and byproducts were further inferred when combined with the mass spectrum. For example, 2,3-Dihydro-fluoranthene (P1) and 8-Phenyl-1,2-dihydro-naphthalene (P2) were identified in the FLA-Fe(III)-montmorillonite system (Figure S3); 1,2-Dihydro-pyrene or 4,5-Dihydro-pyrene (P3), and 1,10a-Dihydro-pyren-1-ol or 4,10c-Dihydro-pyren-4-ol (P4) were analyzed in the PYR system (Figure S4); 7,12-Dihydro-benzo[a]anthracene (P5) was detected in the BaA system (Figure S5); 4,5-Dihydro-benzo[e]acephenanthrylene (P6) was observed in the BbF system (Figure S6); and 5,6-Dihydro-benzo[ghi]perylene or 6,7-Dihydro-benzo[ghi]perylene (P7) were identified in the BghiP system (Figure S7). According to these byproducts and inferred intermediates, a PAH degradation pathway during EPFR formation was proposed, as illustrated in Figure 3. It is noteworthy that 2-Methyl-biphenyl was also analyzed in the Flu system, but EPR signals were not detected (Figures S2 and S8), which means that the degradation of Flu will not cause EPFR formation. Similar results were also reported in the PHE system under irradiation [51]. It could be seen that the transformation pathway of the organic precursors affected the formation of EPFRs. Delocalization of the π-π bonds in the benzene ring structure is thought to be conducive to electron transfer, but the formation of EPFRs also requires a more stable electron cloud structure. The ring-opening products and their intermediates in the Flu system would result in an uneven distribution of electron cloud density and would not be conductive to EPFR formation [57,58]. When focusing on an actual particle system in the environment rather than the nanoscale particles with smaller size and stronger adsorption, the range of organic precursors with the potential ability to generate EPFRs might be more rigorous. Their transformation pathway could affect the EPFR formation to a great extent and needs further theoretical research.

### 3.3. The Hydrogenation of PAHs and EPFR Formation Mechanism

**Figure 4:**
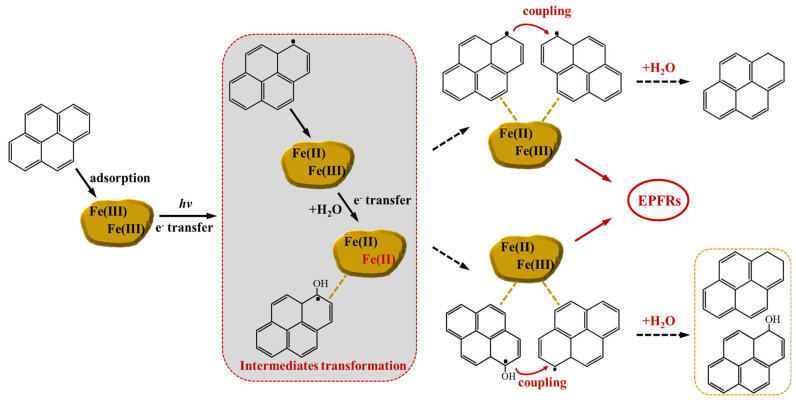
The formation of EPFRs from the hydrogenation of PAHs on simulated Fe(III)-montmorillonite surface under irradiation.

With the information obtained from our results, the proposed pathway for the hydrogenation of PAHs and the EPFR formation mechanism on Fe(III)-montmorillonite particles is outlined in Figure 4. Initially, PYR is adsorbed on the particle surface by interaction with the oxygen of the siloxane (Si-O-Si structure) via hydrogen bonds and the van der Waals force [59,60]. Then, chemisorption occurred under irradiation through one electron transferring from the adsorbed PAHs to the Fe(III) associated with the particles, followed by the formation of intermediates and Fe(II) [36,61,62,63]. For certain PAHs, the electron could be further transferred to form the hydroxylated intermediates in this stage. These intermediates could continue to undergo hydrogenation, inducing the formation of carbon-centered EPFRs. The hydrogenated byproducts were generated with the help of the catalytic role of the mineral particle surface, based on the relevant studies [64,65,66,67]. Specifically, the two radicals could form a C-C bond by connecting the excitation position of both intermediates. Meanwhile, the radical–radical coupling reaction may also occur on the hydroxylated intermediates [68,69,70]. In a water environment, it has been reported that the presence of H_2_O in the mineral particle surface could significantly lower the reaction barrier by forming a hydrogen bond with the precursor and intermediates, which would promote the surface reaction and result in the hydrogenation reaction. Finally, these coupled radical structures broke the bond to generate EPFRs and hydrogenated byproducts in the presence of H_2_O.

### 3.4. Effects of Co-Existing Water Matrix Factors on EPFR Formation

**Figure 5:**
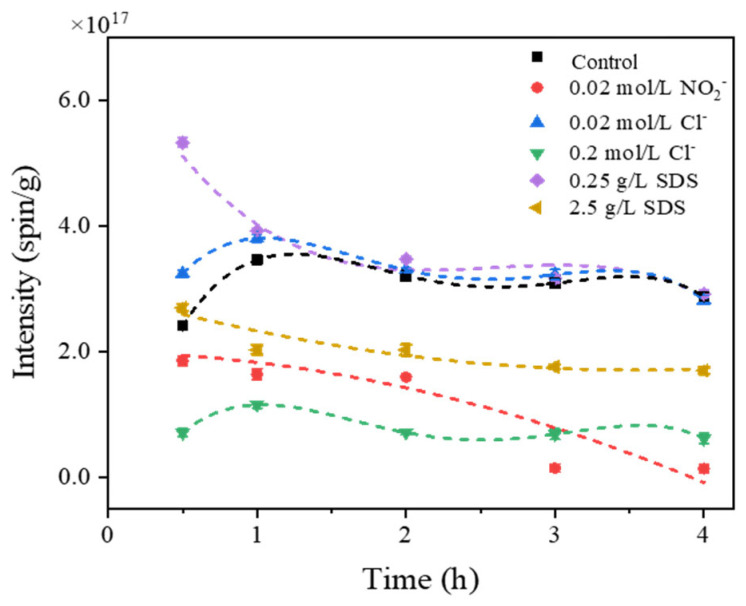
The EPFR intensity trends during the 4 h irradiation in different co-existing water matrix mixed FLA solutions (the linked lines aim to help show the trend).

In terms of the water environment, a variety of potential co-existing water matrix factors naturally exist, and a few studies of their effects on EPFR formation were reported. Typical anions (NO_2_^−^ and Cl^−^) and a surfactant (TWEEN^®^ 80 and SDS) were selected according to their possible effects on the indirect photoreaction of organic precursors and the stability of adsorption between the particles and organic precursors. As shown in Figure 5, the formation of EPFRs was inhibited at a high concentration level of Cl^−^ (0.2 mol/L) anion and showed no significant change at a low concentration (0.02 mol/L). A plausible explanation could be derived from the reaction of Cl^−^ with photoexcited PAHs (Equations (1) and (2)). Cl^−^ consuming ^3^(PAH)^*^ to generate Cl^−^ and Cl_2_^−^ might result in fewer PAH intermediates for EPFR formation. As for the NO_2_^−^ anion, it absorbs light and produces active species as well (Equations (3)–(6)), which should promote the phototransformation; however, a stronger inhibitory effect was found, and EPFRs were not detected even at a high concentration level (0.2 mol/L). We assumed that the formation of hydroxide ions (**·**OH) might interfere greatly with the hydrogenation of PAHs.

Cl^−^ + ^3^(PAH)^*^ → ^3^(PAH-Cl)^*−^ → PAH + Cl^−^ (1)

Cl^−^ + ^3^(PAH-Cl)^*−^ → ^3^(PAH-Cl-Cl)^*2−^ → PAH^−^ + Cl_2_^−^ (2)

NO_2_^−^ + *hv* → **·**NO + **·**O^−^ (3)

**·**O^−^ + H_2_O → **·**OH + OH^−^ (4)

**·**OH + NO_2_^−^ → **·**NO_2_ + OH^−^ (5)

2NO_2_^−^ + *hv* + H_2_O → **·**NO + **·**NO_2_ + 2OH^−^ (6)

The addition of a nonionic surfactant (TWEEN^®^ 80) also caused no EPFRs to be generated at either of the concentration levels. It is generally believed that the nonionic surfactant is adsorbed by hydrogen bonds with the polar groups on the surface of clay particles. The adsorption would cause the interfacial tension to reduce and the particles to be more dispersed in water. In addition, a layer of water molecule film would coat the outside surface of the particles with the hydrophilic groups of the nonionic surfactants. Thus, the EPFR formation was inhibited by reducing the adsorption capacity of the particles in the water. However, the anionic surfactant (SDS) in the water promoted the EPFR formation at a low concentration level (0.25 g/L) and acted as an inhibitor at a high concentration level (2.5 g/L). The negative charge on the surface of sodium-activated montmorillonite is unfavorable to the adsorption of anionic surfactants. The anion exchange adsorption is the main adsorption path between the SDS and clay particles; it increased the adsorption of the organic precursors on the particles, and it might promote the EPFR formation. When more SDS was added to the water, the hydrophobic adsorption would occur between the SDS in the water and the SDS adsorbed on the particles through C-H bonds. The new adsorbed SDS layer changes the hydrophobicity to the hydrophilicity of the particles, which is not conductive to the adsorption of PAHs. Other characteristics of EPFRs are listed in Table S4. These results suggested that effects on EPFR formation caused by co-existing water matrix factors should be considered and need further investigation.

## 4. Conclusions

In this study, we demonstrated that actual particles from contaminated sites could generate EPFRs in an aqueous solution under irradiation. This result is important for understanding the sources and formation mechanisms of EPFRs in water environments. It was generally determined that EPFRs generated in water last at least tens of hours or longer and that a higher concentration level of organic pollutants in the particles is needed for EPFR formation compared with those in the soil or in the air, indicating that EPFRs are harder to generate in water. However, the water environment should also be included as an indispensable medium when studying the migration and transformation of EPFRs in the environment.

This study found that the PAH was a main component contributing to the formation of EPFRs. The interaction between PAHs and particles and their phototransformation made contributions to the EPFR formation in water. Similar mechanisms of the EPFR formation have been studied in previous studies [23,28,29], but the hydrogenation of PAHs was observed in simulated Fe(III)-montmorillonite samples. We assumed that the catalysis of the simulated samples and the interaction of the intermediates resulted in the change in the transformation pathway during the EPFR formation. In addition, the hydrogenation of PAHs would be an effective way to detoxify them. Our study may provide new insights into the control of PAH pollution and requires further research in the future.

This study found that co-existing water matrix factors could affect the photoformation of EPFRs. Their possible effects on the indirect photoreaction of organic precursors and the stability of adsorption between particles and organic precursors might be the internal reason, and further investigation is needed. These results enhanced the understanding of the formation mechanism of EPFRs in water. It has to be realized that more types of EPFRs generated by organic precursors other than PAHs should be investigated, and these precursors may interact with each other during the EPFR formation; these could be emerging research topics and could be helpful in understanding the environmental fates of these organic precursors in water.

## Data Availability

The data that support the findings of this study are available from the corresponding author upon reasonable request.

## Supplementary Materials

The following supporting information can be downloaded at: https://www.mdpi.com/article/10.3390/toxics12110796/s1, Figure S1: EPR signals of actual samples under irradiation, Figure S2: EPR signals of Fe(III)-montmorillonite sample with Nap and FLU and lab-prepared aging particles at different concentrations of PAHs (5 mg/kg, 25 mg/kg and 100 mg/kg) under irradiation for 4 h, Figure S3: Mass spectrum of 8-Phenyl-1,2-dihydro-naphthalene and 2,3-Dihydro-fluoranthene, Figure S4: Mass spectrum of 1,2-Dihydro-pyrene or 4,5-Dihydro-pyrene and 1,10a-Dihydro-pyren-1-ol or 4,10c-Dihydro-pyren-4-ol, Figure S5: Mass spectrum of 7,12-Dihydro-benzo[a]anthracene, Figure S6: Mass spectrum of 4,5-Dihydro-benzo[e]acephenanthrylene, Figure S7: Mass spectrum of 5,6-Dihydro-benzo[ghi]perylene or 6,7-Dihydro-benzo[ghi]perylene, Figure S8: Mass spectrum of 2-Methyl-biphenyl, Table S1: Concentrations of typical PAHs (μg/g dw) in sampling sites, Table S2: EPFRs detected on soil particles and atmospheric particulate matter with different concentrations of PAHs, Table S3: Structures, relevant numberings, and substitution sites based on the predictive approaches of the PAHs, Table S4: The g value and line width (ΔHpp) of EPFRs detected on samples in different co-existing water matrix solutions.
