# Supplementary material for: Photoformation of Environmentally Persistent Free Radicals During Phototransformation of Poly-Cyclic Aromatic Hydrocarbons (PAHs) on Particles in an Aqueous Solution: The Hydrogenation of PAHs and Effect of Co-Existing Water Matrix Factors
Source: Toxics. 2024 Oct 31;12(11):796. doi: 10.3390/toxics12110796 (PMC11597975; doi:10.3390/toxics12110796)
Supplement: Supplementary file 1 [file toxics-12-00796-s001.zip › toxics-3185849-supplementary.pdf]

Supplemental Information

Text S1

Target standards including 15 PHA congeners, including acenaphthylene (ACY), acenaphthene (ACE), fluorene (FLU), phenanthrene (PHE), anthracene (Ant), fluoranthene (FLA), pyrene (PYR), benzo[a]anthracene (BaA), chrysene (CHR), benzo[b]fluoranthene (BbF), benzo[k]fluoranthene (BkF), benzo[a]pyrene (BaP), dibenz[a,h]anthracene (DahA), indeno[1,2,3-c,d]pyrene (IcdP) and Benzo[g,h,i]perylene (BghiP), and surrogate standard ACE-d10, and 2,2-Diphenyl-1-picrylhydrazyl (DPPH) standard, were all purchased from Aladdin Co. Ltd. (Shanghai, China). The purities of all the standards were higher than 95%. Also, analytical grade chemicals, including anhydrous sodium sulfate, (Na_2SO_4) (60 mesh), were purchased from Sigma-Aldrich (St. Louis, MO, USA). Neutral silica gel (70–230 mesh) was purchased from Thermo Fisher Scientific Co.

Text S2

Preparation method for simulated Fe(III)-montmorillonite samples

The simulated Fe(III)-montmorillonite samples were prepared as followed: Montmorillonite was cleaned in ultrapure water at a ratio of 1:20 (w/w) (solid/liquid) and stirred for 8 hours. After centrifugation for 6 minutes at a 100 rounds/min speed, 0.5 mol/L sodium acetate buffer was added dropwise until the pH of the suspension was maintained below 6.8 for 2 hours. Then, 0.1 mol/L NaCl solution was added after centrifugation at a 3300 rounds/min speed for a cation exchange on the montmorillonite.

The samples were centrifuged again before being freeze-dried at -4°C for 48 hours. The treated montmorillonite was passed through a 200-mesh sieve, and 0.05 mol/L $FeCl_3$ solution was mixed with the montmorillonite and stirred for 8 hours to prepare the simulated Fe(III)-montmorillonite samples. The samples were immediately freeze-dried and stored in polyethylene bottles. The pH of the actual samples was around 8.80-8.83 and that of the Fe(III)-montmorillonite samples was around 4.65-4.68. Other relevant characterization results were reported in our previous manuscript (Li, X. et al., 2022).

Text S3

A Bruker A200 electron paramagnetic resonance spectrometer (Karlsruhe, Germany) equipped with an ER075 magnet (65 mm pole cap distance) and an ER 4102ST cavity were used for X-band EPR spectroscopic measurements. All the EPR determinations were undertaken at ambient temperature. The instrument parameters were set as follows: center field at 3361 G, X-band microwave frequency of 9.43 GHz, microwave power of 0.20 mW, spectral window of 200 G, modulation amplitude of 3.00 G, modulation frequency of 100 kHz, time constant of 163.84 ms, conversion time 40.00 ms, sweep time 40.96 s, and the resolution in the X axes of 1024 points and 5 times that of the X-scans. The quantification of the EPFRs was calculated with the help of DPPH. By comparing the DI/N, as calculated from the double integration of the first derivative signal divided by the normalized constant, the concentrations of EPFRs were normalized to the weight (shown in spins/g). The spin number is calculated based on

Commented [EE1]: Please check that the intended meaning has been retained.

these equations:

$$S_{EPFRs} = S_{DPPH} \times \frac{(DI/N)_{EPFRs}}{(DI/N)_{DPPH}} \quad (1)$$

$$\text{Or } S_{EPFRs} = S_{DPPH} \times H_{EPFRs}(\Delta H_{EPFRs})^2/H_{DPPH}(\Delta H_{DPPH})^2 \quad (2)$$

Here, S_{DPPH} is the total spin number of the DPPH spin. $(DI/N)_{EPFRs}$ and $(DI/N)_{DPPH}$ were obtained by double integration of the first derivative signal divided by the normalized constant. H_{EPFRs}, ΔH_{EPFRs} and H_{DPPH}, and ΔH_{DPPH} were the height and width of the EPR signal peaks obtained from the particle samples and DPPH. All the parameters were estimated using Bruker's WINEPR software.

Text S4

We extracted the actual samples (1 g) spiked with surrogate standards in a total of 10 ml of solvent mixture (1:1 (v/v), acetone and dichloromethane) for 30 min in the presence of ultrasound and then centrifuged at 8000 rounds/min speed for 5 min to separate the supernatant. The extracts were collected and repeated three times to ensure the organic compounds were fully extracted. All the extracts were concentrated to 2mL by N_2 stream, and the solvent was exchanged for n-hexane before cleanup. A silica gel column containing silica gel (3% deactivated) and anhydrous sodium sulfate was used to clean the extracts. The eluent was 10 mL of n-hexane and 25 mL of n-hexane/dichloromethane (1:1, v/v), sequentially. Then, the fractions were concentrated to near dryness under a gentle flow of N_2 stream, and the final residue was redissolved in 1 ml hexane. The internal standard hexamethylbenzene was spiked in the final extract for GC-MS analysis. As for the simulated samples, the illuminated sample was

centrifuged and 5 ml of solvent mixture of acetone and dichloromethane (1:1, v/v) was added for ultrasonic extraction. The suspension was collected and repeated three times. After being concentrated to near dryness and redissolved in 1 mL of hexane, the eluates were filtered using a 0.22 μm organic membrane syringe filter (Jin Teng) before analysis. Control experiments were conducted using the same method mentioned above, and the extraction efficiencies of the samples were around 88.57%±2.34% and 82.44%±6.49%, respectively.

Text S5

The photoconversion byproducts were identified by a Q Exactive GC Orbitrap GC-MS/MS (Thermo Fisher Scientific, 2021AAEE, America) operated in a full scan mode (30-500 amu). The oven temperature was programmed starting at 80 °C for 3 minutes, increased to 290 °C at 10 °C/min, and held for 5 minutes.

Table S1. Concentrations of typical PAHs (μg/g dw) in sampling sites.

PAHs	Ring number	Concentration range (μg/g)	Standard deviation (μg/g)	Proportion
Nap	2	—	—	—
ACY	2	5.45~41.42	25.43	1.15%
FLU	2	30.00~42.64	8.94	1.79%
Ant	3	8.06~9.91	1.30	0.44%
FLA	3	80.49~104.91	17.27	4.57%
PYR	4	135.57~137.87	1.63	6.74%
BaA	4	388.99~480.13	64.44	21.41%
BbF	4	265.08~612.86	245.92	21.63%
BkF	4	23.42~83.41	42.42	2.63%
BaP	5	371.71~521.24	105.73	22.00%
IcdP	5	38.07~153.59	81.69	4.72%
DahA	5	62.12~93.61	22.27	3.84%
BghiP	6	111.42~257.65	103.41	9.09%

Proportion column is the relative amounts of the average concentrations

Table S2. EPFRs detected on soil particles and atmospheric particulate matter with different concentrations of PAHs.

Particle Sources	Contaminants	Concentrations	EPFR intensities
Soil particles	∑PAHs	1058.1 mg/kg	$1.25\sim3.58\times10^{16}$ spin/g
Soil particles	∑PAHs	4.755-17.422 mg/kg	$\sim3\times10^{17}$ spin/g
Soil particles	∑PAHs	hundreds to thousands of mg/kg	$10^{17}\sim10^{18}$ spin/g
Soot	∑PAHs	169.6 ± 2.1 mg/kg	$3.20\times10^{17}\sim3.10\times10^{19}$ spin/g
Soil particles (montmorillonite)	Ant	0.386~0.703 mg/g	$10^{17}\sim10^{18}$ spin/g
Soil particles (montmorillonite)	PYR	1.130-~2.441 mg/g	$10^{17}\sim10^{18}$ spin/g
$PM_{2.5}$	∑PAHs	1.09~76.24 ng/m^3	$1.13\sim8.97\times10^{15}$ spin/m^3
$PM_{2.5}$	∑PAHs	74.94 ng/m^3	2.31×10^{13} spin/m^3

Table S3. Structures, relevant numberings, and substitution sites based on the predictive approaches of the PAHs.

PAHs	Structures	HOMO	pp-π	FF	DD
FLU	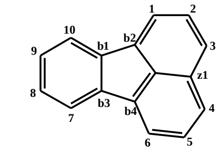	C3,Cb4	Cb1,C3	C3,Cb4	Cb4,C8
PYR	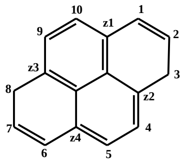	C1,C4	C1,C4	C1,C4	C1,C4
BaA	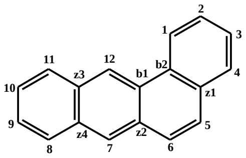	C7,C12	C7,C12	C7,C12	C7,C6
BbF	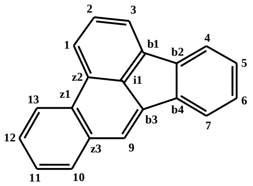	C4,C1	C4,C2	C4,C1	C4,Cil
BghiP	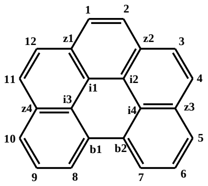	C5,C4	C7,C5	C7,C5	C5,C1

Table S4. The g value and line width ($\Delta H_{p\text{-}p}$) of EPFRs detected on samples in different co-existing water matrix solutions.

	g factor	△Hp-p (Gauss)
Blank	2.00224±0.0000794	10.2151±0.4334
0.02 mol/L NO_2^-	2.00250±0.000362	9.8730±0.3386
0.02 mol/L Cl^-	2.00207±0.0000685	10.70378±0.7205
0.2 mol/L Cl^-	2.00264±0.000235	9.9707±0.8522
0.25 g/L SDS	2.00198±0.0000479	10.8015±0.5837
2.5 g/L SDS	2.00253±0.000206	13.9297±1.099

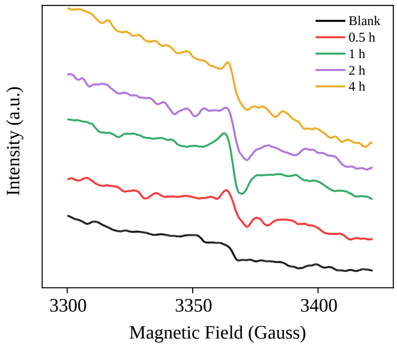

Figure S1. EPR signals of actual samples under irradiation.

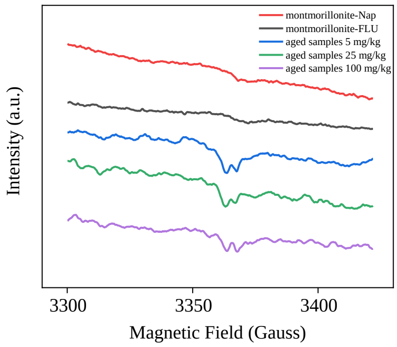

Figure S2. EPR signals of Fe(III)-montmorillonite sample with Nap and FLU and lab-prepared aging particles at different concentrations of PAHs (5 mg/kg, 25 mg/kg and 100 mg/kg) under irradiation for 4 hours.

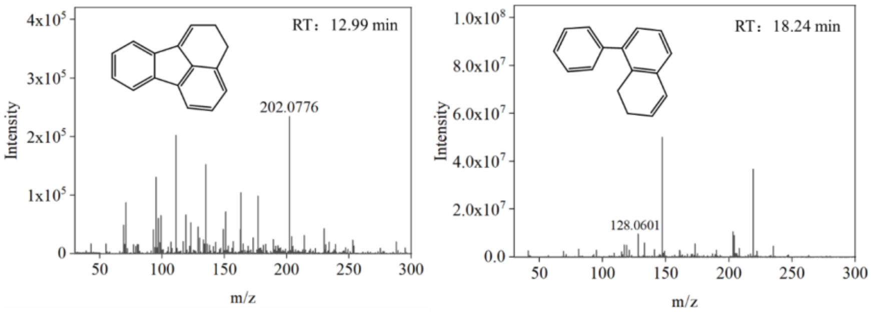

Figure.S3. Mass spectrum of 8-Phenyl-1,2-dihydro-naphthalene and 2,3-Dihydro-

fluoranthene.

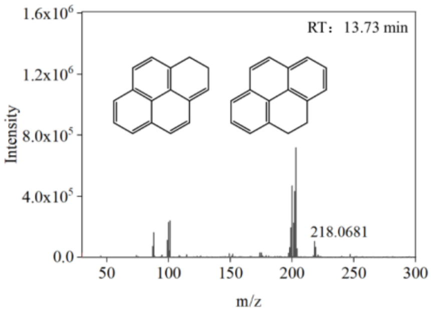

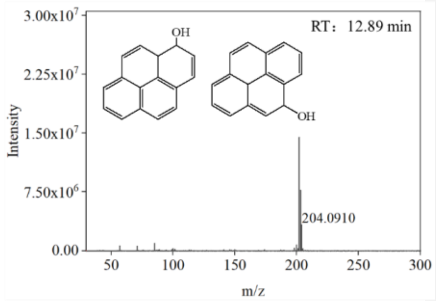

Figure.S4. Mass spectrum of 1,2-Dihydro-pyrene or 4,5-Dihydro-pyrene and 1,10a-Dihydro-pyren-1-ol or 4,10c-Dihydro-pyren-4-ol.

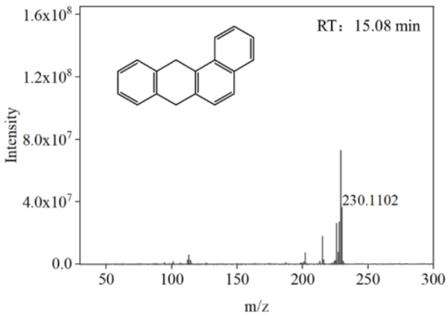

Figure.S5. Mass spectrum of 7,12-Dihydro-benzo[a]anthracene.

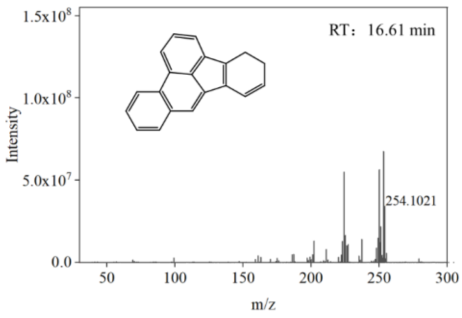

Figure.S6. Mass spectrum of 4,5-Dihydro-benzo[e]acephenanthrylene.

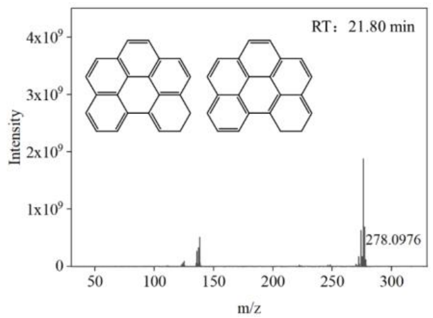

Figure.S7. Mass spectrum of 5,6-Dihydro-benzo[ghi]perylene or 6,7-Dihydro-benzo[ghi]perylene.

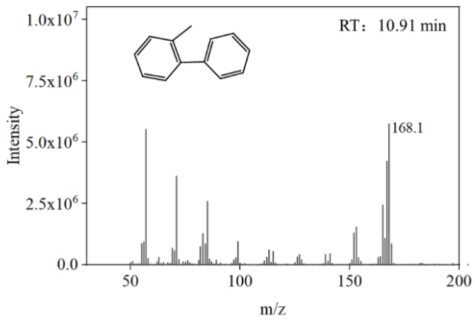

Figure.S8. Mass spectrum of 2-Methyl-biphenyl.
